# Differential landscape of non-CpG methylation in embryonic stem cells and neurons caused by DNMT3s

**DOI:** 10.1038/s41598-017-11800-1

**Published:** 2017-09-12

**Authors:** Jong-Hun Lee, Sung-Joon Park, Kenta Nakai

**Affiliations:** Human Genome Center, the Institute of Medical Science, the University of Tokyo, Tokyo, Japan

## Abstract

Methylated non-CpGs (mCpH; H means A, C, and T) have emerged as key epigenetic marks in mammalian embryonic stem cells (ESCs) and neurons, regulating cell type-specific functions. In these two cell types, mCpHs show distinct motifs and correlations to transcription that could be a key in understanding the cell type-specific regulations. Thus, we attempted to uncover the underlying mechanism of the differences in ESCs and neurons by conducting a comprehensive analysis of public whole genome bisulfite sequencing data. Remarkably, there were cell type-specific mCpH patterns around methylated CpGs (mCpGs), resulted from preferential methylation at different contexts by DNA methyltransferase (DNMT) 3a and 3b. These DNMTs are differentially expressed in ESCs and brain tissues, resulting in distinct mCpH motifs in these two cell types. Furthermore, in ESCs, DNMT3b interacts with histone H3 tri-methylated at lysine 36 (H3K36me3), resulting in hyper-methylation at CpHs upon actively transcribed genes, including those involved in embryo development. Based on the results, we propose a model to explain the differential establishment of mCpHs in ESCs and neurons, providing insights into the mechanism underlying cell type-specific formation and function of mCpHs.

## Introduction

DNA methylation, the addition of a methyl group on the fifth carbon at cytosine, is one of the most important epigenetic modifications. It preferentially occurs at CpG (cytosine followed by guanine) sites in mammalian cells, regulating cell development and maintenance^1, 2^. In contrast, the methylation at non-CpG (CpH; H includes A, C, and T) sites had been barely detected in mammalian cells. However, recent improvements in sequencing technology enabled researchers to determine that significant amounts of methylated CpHs (mCpHs) exist in mammalian pluripotent stem cells and non-dividing cells (i.e., neurons). The mCpHs are involved in cell type-specific regulation such as embryonic stem cell differentiation or synaptogenesis^3–8^. Thus, mCpH, as mCpG, has emerged as a key epigenetic factor, especially in pluripotent stem cells and neuron.

In these cells, both mCpGs and mCpHs are induced by DNA methyltransferase3a and 3b (DNMT3a and DNMT3b, respectively), with allosteric cooperation of DNMT3l^9^. Since these methyltransferases show much higher affinities at CpGs than CpHs, the mCpHs are spatially dependent on mCpGs^3, 6, 10–13^. Despite the dependency, mCpHs regulate several cell type-specific functions independently to the methylation at CpGs. In the brain, mCpHs gradually increase with age, similar with the progression of synaptogenesis^5^; whereas mCpGs do not. In addition, the methyl-CpG binding protein 2 (MeCP2), the mutation of which causes Rett syndrome, binds to not only mCpGs, but also mCpHs^14^. Considering that postnatal onset of Rett syndrome coincides with the emergence of mCpH in neurons, MeCP2-related neuro-diseases could be governed by mCpHs rather than mCpGs^15^. Additionally, in ESCs, the reduction of mCpHs leads to the decrease of differentiation capacity^16^. Thus, even though mCpHs are spatially correlated to mCpGs, they play important roles on cell type-specific processes independently of mCpGs.

One of the underlying mechanisms by which mCpHs govern cell type-specific processes is their differential distribution between the cell types. For example, the CpH methylation primarily occurs at CAG motif in ESCs^8, 17^, while at CAC motifs in neurons^5, 18^, implying that at least two distinct mechanisms for formation (and/or function) of the mCpHs exist^10^. In addition, CpHs tend to be hyper-methylated in actively transcribed genes in ESCs, while hypo-methylated in neurons^5, 8^. Since DNA methylation tends to repress gene expression in most cell types^19^, the positive correlation between CpH methylation and gene expression in ESC has been an enigma among researchers^8, 10^. Thus, mCpH shows different distribution and potential role in gene expression between ESCs and neurons. However, what causes the differences remains unknown.

In this study, to uncover the mechanism of the differential CpH methylation, we analysed the whole genome bisulfite sequencing (WGBS) data of human ESCs, neurons, and brain tissues. In addition, we included WGBS data of DNMT knockout human and mouse ESCs to investigate the role of DNMTs on cell type-specific methylation^5, 12, 20–25^. Through a comprehensive analysis, we found that CpH methylation pattern near CpGs (within ± 100 base pair (bp) from CpGs) is highly distinguishable between ESCs and neurons. Further analyses uncovered that DNMT3a and DNMT3b, differentially expressed in those two cell types, preferentially methylate cytosine in different contexts, resulting in distinct motifs and patterns of mCpHs in ESCs and neurons. Additionally, in ESCs, the DNMT3b preferentially interacts with H3K36me3, resulting in hypermethylation at CAGs on actively transcribed gene-body regions. The methylated CAGs were enriched in genes related to embryo development, indicating possible role of mCpHs on embryogenesis. Based on these results, we suggest a differential CpH methylation model that could explain distinct features and functions of mCpHs in ESCs and neurons. Altogether, our results provide new insights on cell type-specific formation and function of mCpHs in mammals.

## Results

### The integrative approach for WGBS read-aligning successfully reproduced known characteristics of mCpH in ESCs and neurons

To analyse methylation in both CpG and CpH contexts, we aligned WGBS reads generated from various experiments (S. Table 1). Three bisulfite read aligners, Bismark^26^, BSMAP^27^, and BS-seeker2^28^, were used for read aligning, and the outputs were integrated as previously described^29^. This integration method has proven to improve methylation detection accuracy and reduce artifacts from experimental settings^29^.

**Figure 1:**
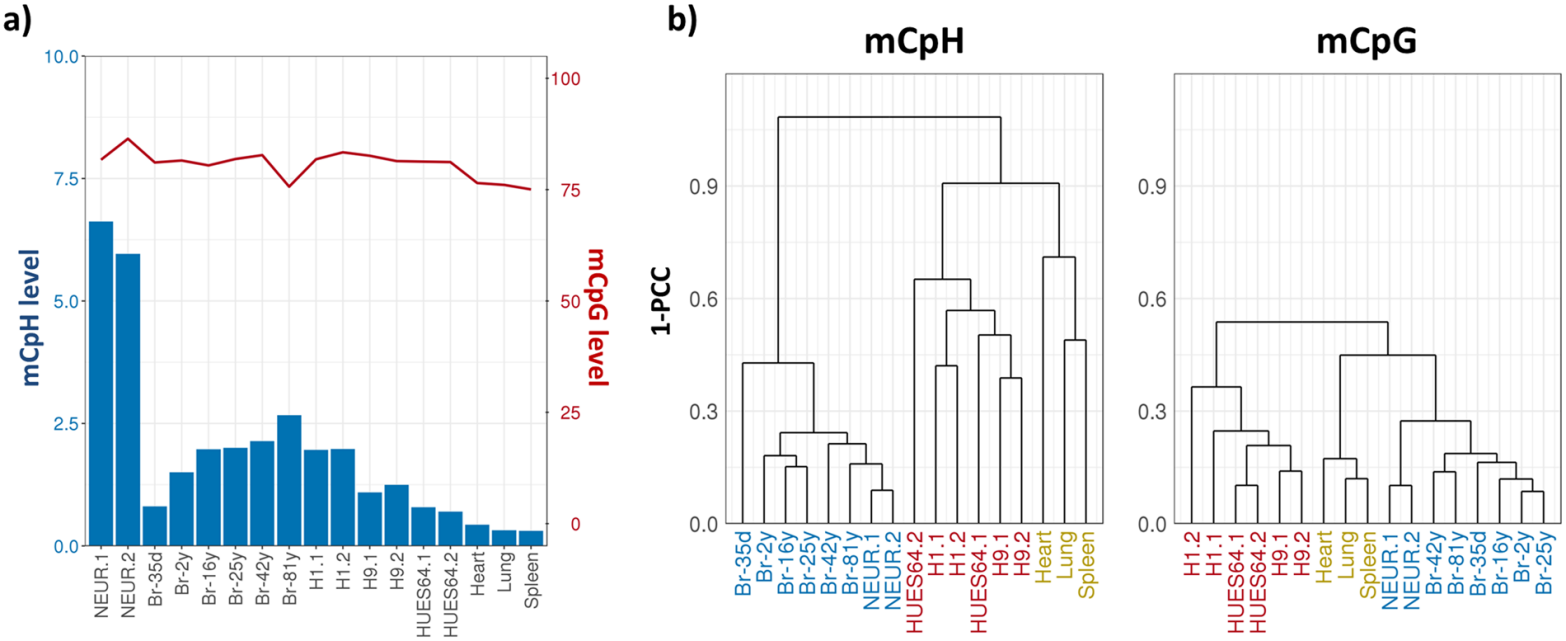
Human methylome from integrated WGBS data. (**a**) Genome-wide average methylation level at CpGs (red line) and CpHs (blue bar) extracted from WGBS data. NEUR and Br represent neurons and brain tissues, respectively. The numbers followed by Br- means age of samples (d and y mean day and year, respectively). H1, H9, and HUES64 are human embryonic stem cell lines. Numbers at the end of the labels represent biological replicates. (**b**) WGBS samples hierarchically clustered by genome-wide CpG (left) and CpH (right) methylation levels. The whole genome was divided into 1 k-bp-long blocks and the average methylation levels of blocks were used to calculate the Pearson’s correlation coefficient (PCC). Colours in the x-axis represents the same cell (or tissue) types (blue: brain and neurons, red: ESCs, and yellow: other tissues).

Statistic features of the regenerated data coincided with those obtained in previous studies (Fig. 1a)^3, 5–8, 10^. In human samples, although most of the CpGs were hyper-methylated (75–85%), the average methylation levels at CpHs (mCpH level) were distinct across cell types. It was mostly abundant in neurons (>5%), abundant in ESCs derived from male (H1) and matured brain tissues (>1%), detectable in ESCs derived from female (H9), early-passage ESCs (HUES64), and immature brain tissues (0 to 5 year-old; about 1%), and mostly undetectable in other tissues (The heart, spleen, and lung; <0.5%). In addition, we observed an increase of mCpH levels along with brain aging^5^, and lower mCpH level in H9 than in H1, as previously reported^8, 17, 30^. Remarkably, the WGBS samples were clearly clustered based on the tissue of origin (or cell types) by both CpG and CpH methylation patterns, indicating our integrated dataset well represents differential methylation pattern among cell types (Fig. 1b).

Lastly, we confirmed the known difference of mCpHs between ESC and neuron. The motif abundant at hyper-methylated CpHs (${{Me}}_{i}$>0.5, See Method section) was “CAG” in all ESC samples, and “CAC” in all neuron and brain samples (S. Figure 1a), as previously reported^5, 6, 8, 17, 18^. In addition, the mCpH level in gene-body regions was positively correlated to FPKMs in ESCs (H1 and HUES64), whereas it was negatively correlated in adult brain (S. Figure 1b, Spearman’s rank correlation coefficient (ρ) equals to 0.37, 0.3, and −0.29, respectively).

Altogether, our integrated methylome successfully reproduced the known characteristics of CpH methylation in mammalian cells. Since the mCpH levels in early brains (Br-35d and −2y) and somatic cells are extremely low, we excluded these samples from further analysis and focused on methylation in ESCs, neurons, and adult brains. In addition, we considered that the characteristic of mCpHs in brain samples represents that in neurons, since mCpHs are mostly absent in other cells of brain^5^.

### Cell type-specific CpH methylation patterns are observed in mCpG-proximal region (±100 bp)

**Figure 2:**
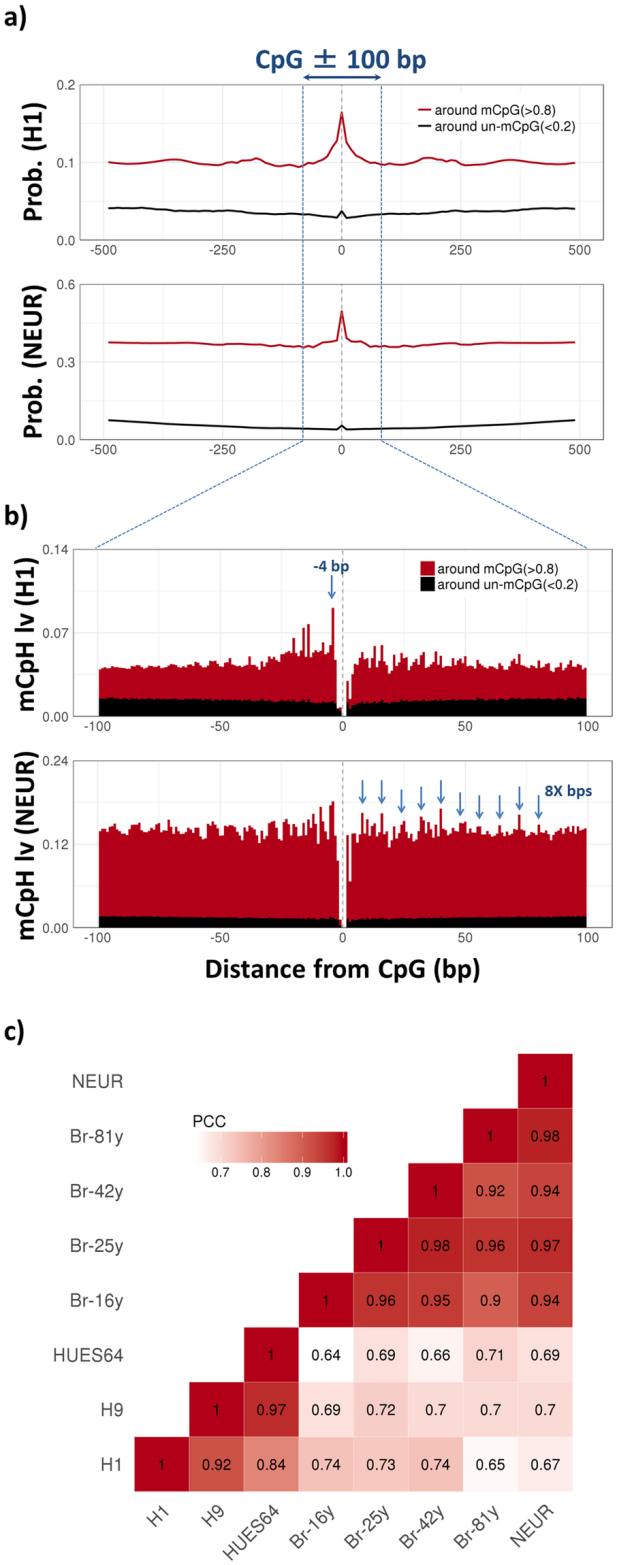
Cell type-specific CpH methylation around CpGs. (**a**) Probability that mCpHs exist around methylated CpGs (metylation level >0.8, red), and un-methylated CpGs (methylation level <0.2, black). The identification of (un)methylated CpHs is described in the Method section. To calculate the probabilities, the surrounding (±500 bp) of every CpG divided into 10-bp-long blocks, and blocks containing more than one mCpH were counted, given the condition of CpGs (methylated or un-methylated). H1 represent H1 sample replicate 1 (H1.1), and NEUR represents neuron sample replicate 1 (NEUR.1). (**b**) The accumulated CpH methylation levels at each distance from methylated CpGs (red bars), and un-methylated CpGs (black bars). Blue arrows highlight mCpH level peak at -4 bp from mCpG in ESC, and 8 bp-periodicity of the peaks in neuron. (**c**) Heatmap showing the Pearson’s correlation coefficients (PCC) across mCpG-proximal (±100 bp) mCpH levels of the samples. H9 represents H9 replicate 1 (H9.1), HUES64 represents HUES64 replicate 1 (HUES64.1).

Using the WGBS dataset, we analysed correlation between CpG and CpH methylation. We divided human whole genome (hg19) into 1 k-bp blocks and compared average methylation level at CpGs and CpHs in each block. The genome-wide CpG and CpH methylation levels were positively correlated as previously reported^3, 6, 11, 18^ (S. Figure 2a; Pearson’s correlation coefficients (PCC) were from 0.27 to 0.44). In further analysis with blocks in which CpGs are hemi-methylated (difference of mCpG level between DNA strands > 0.5), mCpH levels were significantly higher at the same strand with highly methylated CpGs, compared to those at the opposite strand, indicating that CpG and CpH methylation correlated in a strand-specific manner (S. Figure 2b,c). Additionally, the correlation between mCpG and mCpH was significantly higher at exons, promoter regions, and putative enhancer regions, implying that the co-regulation of CpG and CpH methylation mainly occurs in transcription-regulatory regions (S. Figure 2d).

**Figure 3:**
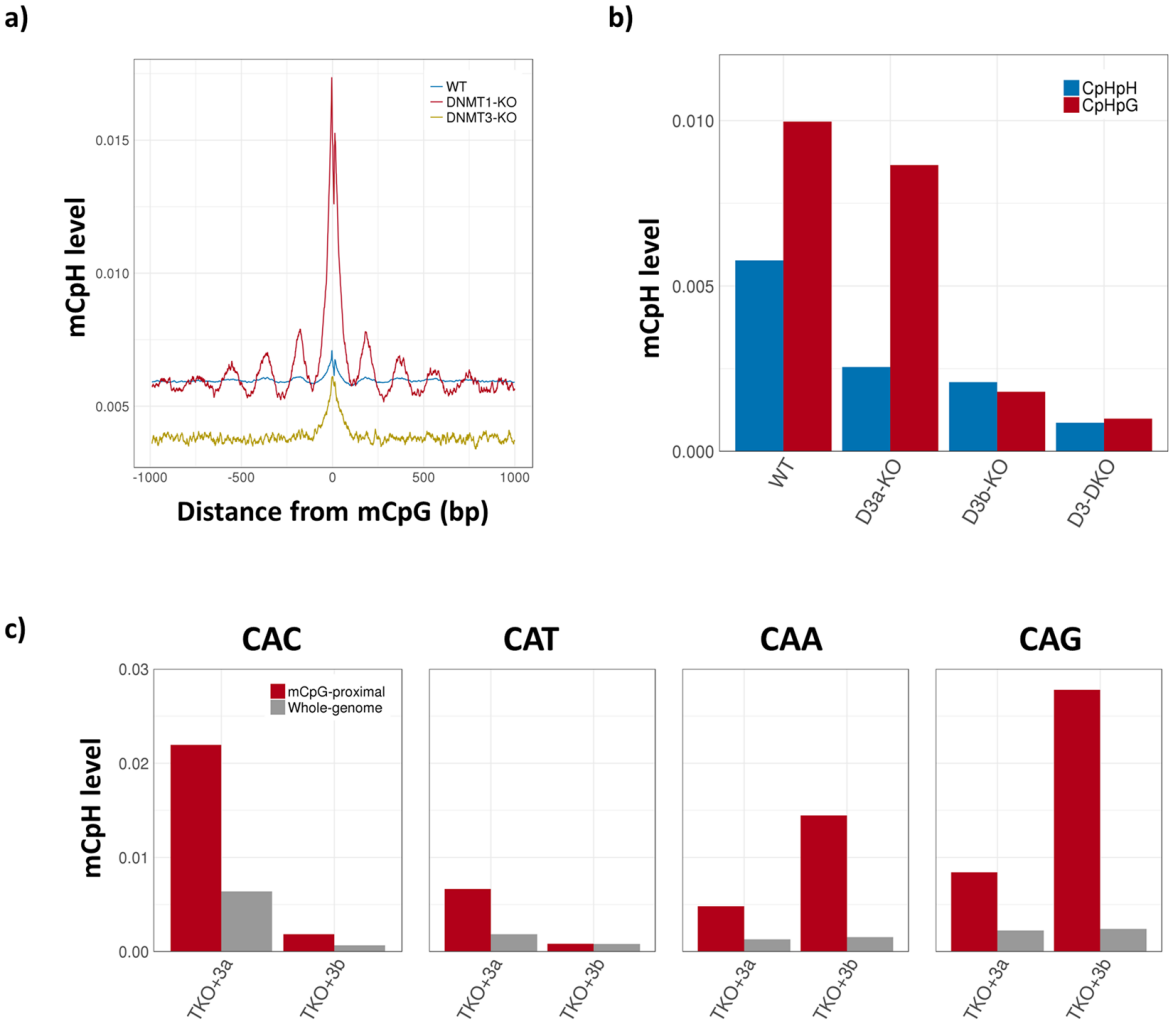
Methylation at cytosines in different contexts by DNMT3a and DNMT3b in mouse (**a**,**c**) and human (**b**) ESCs. (**a**) The window-slided (window size = 10 bp) average CpH methylation levels around mCpG (methylation level >0.8) in WT, DNMT1-KO, and DNMT3-KO mouse ESC. (**b**) Bar graphs showing the average methylation levels at CpHpH (blue) and CpHpG (red) contexts in wild type (WT), DNMT3a-knock out (D3a-KO), DNMT3b-knock out (D3b-KO), and DNMT3a/b-knock out (D3-DKO) HUES64 cell line. WT ESC is HUES64.2. (**c**) Average methylation level at CAC, CAT, CAA, and CAG contexts in mouse ESCs, in which DNMT1, DNMT3a, and DNMT3b were knocked out (TKO), and DNMT3a and DNMT3b were reintroduced (TKO+3a and TKO+3b), respectively^24^. Red and grey bars respresent average mCpH level in mCpG-proximal regions (mCpG level>0.5) and whole genome, respectively.

Next, we attempted to measure distance required so that mCpG and mCpH could be co-regulated. First, we measured the probability that mCpHs appear at each distance from methylated or un-methylated CpGs (Fig. 2a). The probability showed a clear peak near mCpGs in both ESCs and neurons, indicating the concentration of mCpHs near mCpGs. The tendency was confirmed with a subset of CpGs, around which (±500 bp) no other CpGs exist (S. Figure 3a). Second, we measured the correlation between mCpGs and surrounding mCpHs. The correlation coefficients were also significantly higher near CpGs (S. Figure 3b) supporting that CpG and CpH methylation could be co-regulated when these are closely positioned. Thus, we concluded that methylation at CpHs is highly dependent to that at proximal CpGs, when the distance between CpGs and CpHs is less than ± 100 bp, which sufficiently covers the probability and correlation coefficient peaks.

We analysed methylation pattern at CpHs within ± 100 bp from mCpGs (Fig. 2b). In ESCs, a clear CpH methylation peak was observed at −4 bp from mCpG as previously reported^31^. On the other hand, in neurons and brains, there was an 8–10 bp periodicity among mCpH peaks starting from mCpGs. This has been suggested as a mark for CpG methylation by DNMT3a-DNMT3l enzyme complex^32^, indicating that mCpGs and mCpHs are co-regulated by DNMT3a in neurons and brain tissues. Remarkably, the methylation pattern at mCpG-proximal CpHs was conserved among same cell types but clearly distinguishable between ESCs and neurons (Fig. 2c), implying that different methylation mechanisms exist between the two cell types. Altogether, our data indicated that the CpHs are methylated along with CpGs, but show different methylation patterns in ESCs and neurons.

### DNMT3a and DNMT3b preferentially methylate CAC and CAG contexts, respectively

To uncover what causes the distinct methylation patterns at mCpG-proximal CpHs, and the known differences of mCpHs in ESC and neuron (S. Figure 1a and b), we analysed DNMT knockout human and mouse ESCs.

**Figure 4:**
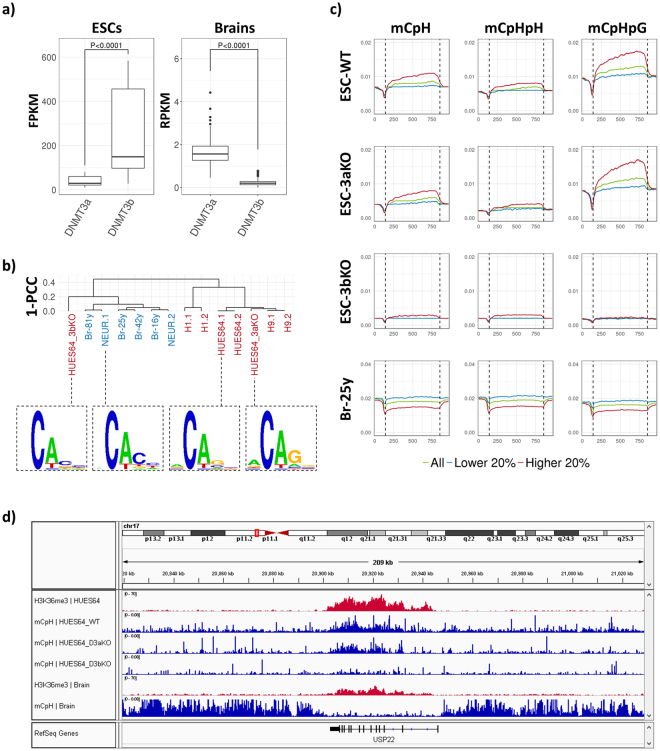
Cell-type specific features of mCpHs induced by differential activity of DNMT3s. (**a**) Boxplot representing the FPKM and RPKM of DNMT3a and DNMT3b in 36 ESCs (H1 and H9) and 143 adult brain tissues (from 13 to 40 year-old) of human. The boxes include values from 25% to 75% in order. The P-value was calculated from Wilcox-rank t-test. Specifics for the data are described in S. Table 2. (**b**) Hierarchical clustering results by mCpG-proximal CpH methylation pattern. Shown in bottom represents abundant DNA motif near mCpHs (−1 bp to 4 bp) in mCpG-proximal region. (**c**) Methylation levels around highly expressed (red, higher 20%), lowly expressed (blue, lower 20%), and total (yellow) genes. The gene bodies and surround (±20% of those) were normalized into 1k-bins and extracted the average methylation levels at each bin, and processed by sliding window (window size = 30 bp). The transcription star site (TSS) and transcription terminate site (TTS) are in 142’th and 857’th bins, respectively, described as black dotted lines. (**d**) H3K36me3 peaks and mCpH levels around gene-body region of ubiquitin specific peptidase 22 (USP22), an epigenetic modifier that regulates embryonic stem cell differentiation^60^. This is printed by IGV^61^.

As a first step, we analysed mouse ESCs (mESCs) in which DNMT1, and both DNMT3a and DNMT3b are knocked out (D1-KO and D3-KO mESC, respectively)^23^. The mCpH level was largely decreased in D3-KO mESC, indicating that DNMT3a and DNMT3b are mainly responsible for CpH methylation (S. Figure 4a). Interestingly, in D1-KO mESC, the mCpH level was higher near mCpGs, and showed clear peaks with 180 bp interval, close to the nucleosome positioning pattern (Fig. 3a). Since both mCpGs and mCpHs are mainly introduced by DNMT3s in D1-KO sample, this result indicates that CpHs are methylated along with CpGs, by DNA walking of DNMT3s. In addition, the correlation between mCpG and mCpH levels was higher in D1-KO mESC and lower in D3-KO mESC, compared to that in wild type mESC (S. Figure 4b; PCC = 0.4, 0.07, and 0.2, respectively). Altogether, our data confirmed that DNMT3a and DNMT3b methylate CpHs, in correlated way to the methylation at CpGs.

Next, we analysed the contribution of DNMT3a and DNMT3b to CpH methylation in human ESC (HUES64 cell line). We divided the CpHs into two groups, asymmetric (CpHpH; CpH followed by A, C, or T), and symmetric (CpHpG; CpH followed by G) CpHs^6, 33^. Remarkably, in the DNMT3a knockout sample, the methylation level at CpHpH contexts decreased more than that at CpHpG contexts, whereas the opposite was observed in the DNMT3b-knockout sample (Fig. 3b). Similarly, in DNMT3b-knocked out H9 cells^20^, the methylation level at CpHpG contexts decreased more than that at CpHpH contexts (S. Figure 4c). Further analyses indicated that the methylation level at CAC tri-nucleotides was largely decreased by DNMT3a-knockout, whereas that at CAGs was decreased by DNMT3b-knockout (S. Figure 4d). The methylation level at CAAs was also reduced by the DNMT3b-knockout, even though the methylation level in wild type was not as high as that at CACs or CAGs. The methylation level in other contexts did not show significant difference.

We confirmed similar tendency in mouse ESC samples, generated by Dr. Tuncay Baubec’s group^24^. They knocked out DNMT1, DNMT3a, and DNMT3b from mouse ESCs and then reintroduced DNMT3a and DNMT3b, respectively, to measure *de novo* methylation by each methyltransferase. With this sample set, we confirmed preferential *de novo* methylation at mCpG-proximal CpHs by the DNMTs (Fig. 3c). Remarkably, the cytosines at CACs are more methylated by DNMT3a than by DNMT3b, whereas those in CAGs are mostly methylated by DNMT3b. The methylation at CAAs increased as DNMT3b re-induced in lower level compared to that at CAGs, as shown in human ESC samples. Thus, we concluded that both DNMT3a and DNMT3b are responsible for the methylation at CpHs, especially near mCpGs, but they methylate cytosines in different contexts; DNMT3a primarily methylate CAC tri-nucleotides, while DNMT3b methylates CAG tri-nucleotides.

### The differential activities of DNMT3a and DNMT3b cause distinct features of mCpHs in ESCs and neurons

To understand how the differential activities of DNMT3a and DNMT3b affect cell type-specific features of mCpH, we analysed WGBS, RNA-seq, and ChIP-seq data of human ESCs, neurons, and brain tissues.

First, by combining expression data from 143 human adult brains and 33 human ESCs (S. Table 2), we confirmed that DNMT3b is highly expressed in ESCs, whereas DNMT3a is in adult brains (Fig. 4a). This result is coincided with previous result^34^, reporting that DNMT3b is actively transcribed in early ESCs, but decreased as cell differentiated. Remarkably, the abundant motif at mCpHs in DNMT3b-knockout human ESC was not CAG, but CAC (S. Figure 1a), indicating that the hyperactivity of DNMT3b causes the preferential methylation at CAGs in ESC. In addition, the methylation pattern at mCpG-proximal CpHs in DNMT3b-knockout ESC was more similar to that in the brains and neurons, than that in wild type ESCs (Fig. 4b). Thus, our data revealed that the differentially enriched mCpH motifs in ESCs and neurons, as well as the distinct mCpG-proximal mCpH patterns in the two cell types, are caused by the differential activities of DNMT3a and DNMT3b.

Next, we attempt to understand whether the activity of DNMT3s affects the correlation between CpH methylation and gene expression. In contrast with neurons and brains, the highly transcribed genes in ESC showed higher mCpH level in their gene-body regions (Fig. 4c). This tendency remained in DNMT3a-knockout ESC, however, almost disappeared in DNMT3b-knockout ESC. In addition, the hyper-methylation in highly expressed gene-body regions was greatly focused on CpHpG contexts, at which methylation mainly occurs by DNMT3b. Thus, we concluded that DNMT3b causes the positive correlation between mCpH level and gene expression level in ESCs.

**Figure 5:**
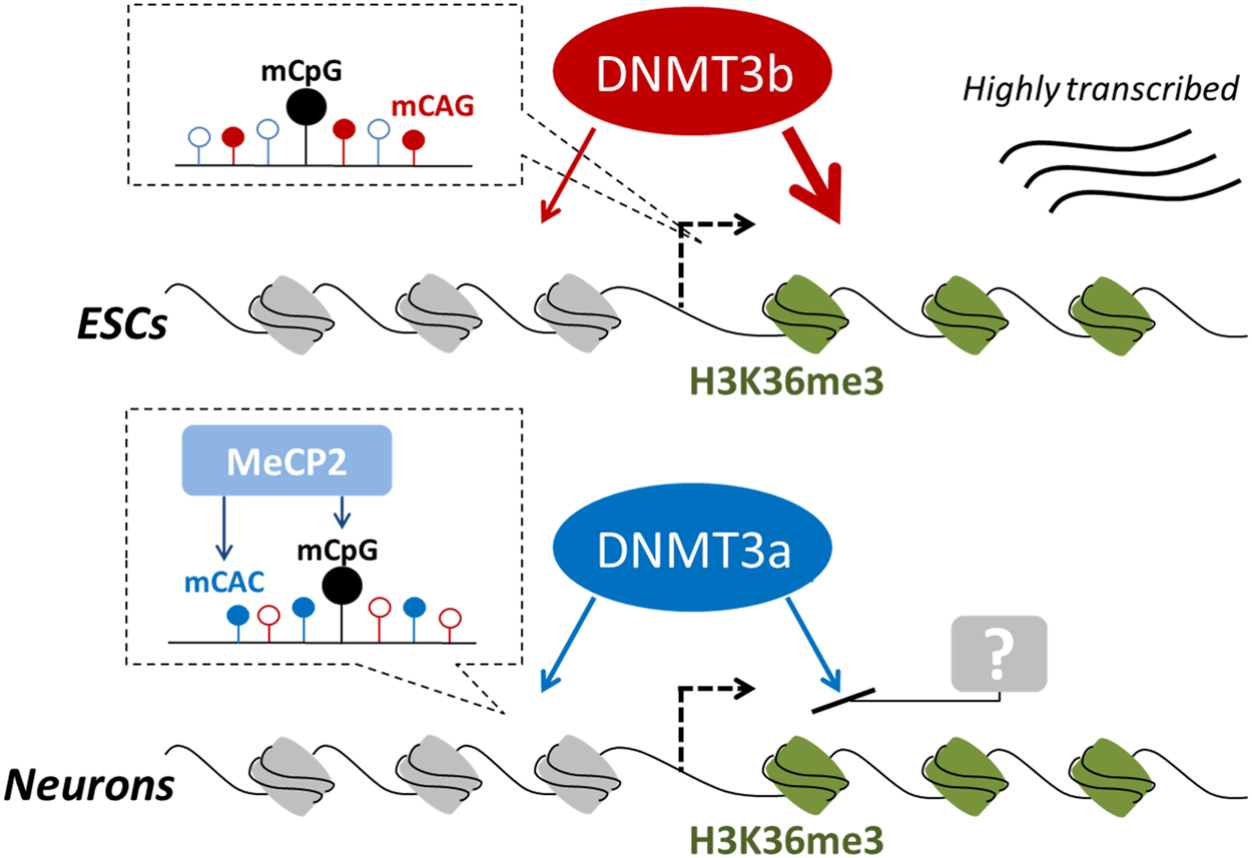
Proposed differential methylation mechanism between ESCs and neurons. The illustration describes two main discovery about differential CpH methylation in ESCs and neurons. First, the DNMT3b is actively transcribed in ESCs, primarily methylating CAG contexts, whereas the DNMT3a is more transcribed in neuron, methylating CAC contexts. The methylated CACs are recognized by proteins related to brain diseases, such as MeCP2^44^. Second, the DNMT3b preferentially interacts with H3K36me3 marks, abundant in actively transcribed gene-body regions, whereas DNMT3a showed little preference. The filled and unfilled circles represents methylated and unmethylated statuses, respectively.

To explore further, we focused on previous reports that the DNMT3b interacts with tri-methylated histone 3 lysine 36 (H3K36me3), enriched in highly expressed genes^24, 35^, assuming that the preferential interaction between DNMT3b and H3K36me3 causes the hyper-methylation at CpHs in highly expressed gene-body regions. To confirm this, we analysed the methylation at CACs and CAGs upon 7 histone modifications, known as markers of transcription regulatory regions such as enhancer, promoter, or gene-body (S. Table 3)^36^. Among the histone marks, the H3K36me3 was highly positively correlated with methylation at CAGs, compared to the methylation at CACs (S. Figure 5a; PCC equals to 0.34 and 0.15, respectively). The positive correlation became weak as DNMT3b knocked out (PCC equals to 0.21). In addition, we confirmed that CpH methylation is more positively correlated with the H3K36me3 overlapping rate (Method section), than with gene expression level (S. Figure 5b; Spearman’s rank correlation coefficient (ρ) equals to 0.73 and 0.33, respectively). Altogether, we concluded that the interaction between DNMT3b and H3K36me3 leads to the positive correlation between CpH methylation and gene expression in ESC.

We confirmed this with mouse ESCs, by analysing the mCpH level and expression level of genes in samples that DNMTs and SET domain containing 2 (SETD2), a catalyser of H3K36me^37^, were knocked out^24^ (S. Figure 5c). The mCpH level tended to be high at actively expressed gene bodies in wild type ESCs (ρ = 0.12), whereas the tendency disappeared as DNMT1, 3a, and 3b are knocked out (Corr. = −0.04). Remarkably, the positive correlation was recovered by reintroducing DNMT3b (Corr. = 0.02), whereas it was not by reintroducing DNMT3a (Corr. = −0.06). The positive correlation in DNMT3b-reintroduced sample disappeared as removing H3K36me3 marks by SETD2-knockout (Corr. = −0.15). Altogether, the results indicated that the positive correlation between CpH methylation and gene expression in ESC is caused by the activity of DNMT3b, preferentially methylates CAGs upon H3K36me3 marks that enriched in actively transcribed gene-body regions.

Lastly, we attempted to explore whether the methylation by interaction between H3K36me3 and DNMT3b is involved in ES-specific functions. We screened 418 genes of which CAGs in gene-body regions are hyper-methylated (*M*_*ei*_> 0.05, top 3% of all genes), and more than half of the gene-body regions are covered by H3K36me3 marks (${histone\; overlapping\; rate}$ > 0.5, S. Figure 6a). The enriched gene ontology terms (GO terms, S. Table 4) of the genes were clustered into several representative biological processes such as mRNA metabolism, chromosome organization, and cellular response to DNA damage stimulus (S. Figure 6b, S. Table 5). These have been identified as being regulated by H3K36me3 marks in previous studies^38–40^, indicating that our data represents the properties of H3K36me3-enriched genes. Remarkably, our gene set included 13 genes related to embryo development (GO:0009790, P-value < 7.54 × 10^−4^, S. Table 6). The gene-body regions of the genes were clearly marked by both H3K36me3 marks and mCpHs in ESC, whereas these were not marked by mCpHs in brain tissue (Fig. 4d, S. Figure 7). The mCpHs disappeared in DNMT3b-knocked out ESC, indicating that these are introduced by DNMT3b. Thus, our data implied that the mCAGs, outcome of the preferential interaction between H3K36me3 and DNMT3b, marks genes involved in embryo development.

## Discussion

Through comprehensive analysis of WGBS data, we uncovered the differential mechanism of CpH methylation in the two representative mCpH-containing mammalian cell types, ESC and neuron^10^.

WGBS has been considered as the only method to extract reliable information about mCpH, since other experiments such as microarray data^41^ or reduced representation bisulfite sequencing (RRBS)^42^ are mainly targeting CpG dinucleotides^43^. However, this method is financially and timely consuming, so that researchers had to deduce results from insufficient number of samples. The integrative read aligning strategy solved this problem by facilitating the use of public data with improved accuracy and reduced experimental artifacts. Through this integrative approach, we employed 21 human WGBSs and 8 mouse WGBSs including neurons, brains, ESCs, DNMT-knockout ESCs, and other somatic cells. The quality of the resulting dataset was verified by reproducing the known characteristics of mCpH in each cell type, and by clustering the samples into their originated cell types by genome-wide methylation pattern. The enlarged sample set by integrating public data, with improved detection quality, contributed to the statistical robustness of the following results.

Through analysing genome-wide methylation pattern, we confirmed that mCpHs are spatially correlated with mCpGs. Especially, these were co-regulated by DNMT3 when the distance between CpG and CpH is under ± 100 bp. This tendency was observed in both ESCs and neurons. Remarkably, however, there were distinct methylation patterns at CpHs in CpG-proximal regions ( ±100 bp from CpGs) between ESCs and neurons. Along with previously reported difference of the mCpHs in ESCs and neurons^10, 31, 32^, this distinct methylation pattern could be a marker for distinguishing the two cell types, and be a measure for the process of ESC differentiation to neuron.

We further explored that the differential distribution of mCpHs is caused by differential activities of DNMT3a and DNMT3b. By comparing genome-wide mCpH level in wild type and DNMT knockout ESCs, we found that DNMT3a preferentially methylates CAC contexts, whereas DNMT3b methylates CAG contexts. The differential targeting of DNMT3a and 3b, combined with their differential expression in ESCs and the brains, is suggested as the main reason of the distinct mCpH distribution in the two cell types. In ESCs, the hyperactivity of DNMT3b results in preferential methylation at CAGs, whereas in neurons, the DNMT3a is highly expressed, resulting in hypermethylation at CACs. Decisively, in DNMT3b-knockout ESCs, mCpH was enriched in CAC context than CAG, indicating that the enriched mCpH motif, CAG, is caused by DNMT3b. Additionally, the mCpG-proximal CpH methylation pattern in DNMT3b-knockout ESC was more similar to that in brain tissues and neurons, than to that in wild type ESCs, implying that the mCpG-proximal mCpH pattern in ESC is induced by the hyperactivity of DNMT3b. On the other hand, in neuron, the hyperactivity of DNMT3a results in preferential methylation at CAC motifs. Considering that MeCP2, a DNA-binding protein that broadly related to neuro-diseases, preferentially binds methylated CACs^44^, the CpH methylation by DNMT3a could be a key regulator of neuron-specific cellular processes. Altogether, we concluded that the differential activity of DNMT3a and DNMT3b induces the distinct distribution of mCpHs in ESCs and neurons. The molecular-level mechanism of the differential targeting by the enzymes remains as further study subject.

Finally, the positive correlation between CpH methylation and gene expression in ESCs was explained by the activity of DNMT3b. In a recent study^24^, DNMT3b showed preferential interaction with H3K36me3 mark in highly expressed gene bodies in ESCs. In our analysis, the methylated CAGs (mCAGs) showed spatial correlation with H3K36me3, and significantly accumulated in highly expressed gene-body region in human ESC. Decisively, the positive correlation between mCpH level and expression level disappeared when either DNMT3b or SETD2, catalysing H3K36me3 marks, was knocked out, indicating that the preferential interaction between DNMT3b and H3K36me3 marks causes the positive correlation between CpH methylation level and gene expression. Additionally, we confirmed that the methylation at CAGs, mediated by the interaction between DNMT3b and H3K36me3, marks genes involved in embryo development. This implies that the DNMT3b- H3K36me3 interaction, and/or the outcome of the interaction could be involved in ES-specific cellular processes. For example, recent study found that mCpGs, mediated by the interaction between DNMT3b and H3K36me3, prevent spurious transcription initiation in mouse ESC^35^. The mCAGs could be involved in fine-tuning of the process by the clearly marking actively transcribed gene-body regions, even though further study is necessary for proving it. On the other hand, in neuron and brain, the methylation at CpHs in gene-body regions could be hindered by other transcription factors (TFs) in same way with that at CpGs^45^, since DNMT3a, actively transcribed in neuron, showed little preference at gene-body regions or H3K36me3 marks^24^.

Based on these results, we suggest a differential CpH methylation mechanism by DNMT3a and DNMT3b in ESCs and neurons, which instructs further research directions (Fig. 5). Altogether, this study uncovered the mechanism underlying the differential distribution and functions of mCpH in ESCs and neurons, and provides insightful information regarding the cell type-specific CpH methylation mechanism.

## Methods

### WGBS data analysis

WGBS data were downloaded from NCBI Gene Expression Omnibus (GEO). We performed quality control by fastqx-toolkit^46^ with two steps. First, we trimmed tails of the reads of which quality score is under 20. Then, we removed reads of which length became less than half after the trimming or the average read quality is still under 20. Then, we mapped the high-quality bisulfite sequenced reads into the reference genomes (hg19 for human samples and mm10 for mouse samples) by three bisulfite-read aligners, Bismark^26^, BSMAP^27^, and BS-seeker2^28^. Then, we removed duplicated reads by using Picard (http://broadinstitute.github.io/picard) (for the output of Bismark and BS-seeker2) and Samtools^47^ (for the output of BSMAP), following the suggestion from the manual of each bisulfite-read aligner. Then, we extracted cytosine positions covered by more than 5 reads, aligned by more than two aligners.

### Methylation detection

Methylation levels at the cytosines were calculated as read-depth weighted average of those from individual aligner^29^. Then, the non-conversion rate was subtracted from the methylation levels at each cytosine, based on the previous statistical model^5^.

1 $$M{e}_{i}=\frac{{{\sum }^{}}_{j}{M}_{ij}}{{{\sum }^{}}_{j}{t}_{ij}}-non\,conversion\,rate$$

Where ${{Me}}_{i}$ is methylation level at cytosine $i$, ${t}_{{ij}}$ is aligned read number at cytosine $i$ by bisulfite-read aligner ${\rm{j}}$, and ${M}_{{ij}}$ is unconverted read number at cytosine $i$ by bisulfite-read aligner ${\rm{j}}$. In case the ${{Me}}_{i}$ is under 0, we set *M*_*ei*_ = 0.

### Identification of methylated cytosines

Since the distribution of the methylation levels at CpGs and CpHs are different, we used different strategy for defining methylated or unmethylated status of the two contexts. The mCpG was defined as $M{e}_{i}\,\geqq \,0.8$. The mCpH, used for calculating the probability that mCpH exists around CpGs (Result 2), was identified by a previously introduced method^6^. We used binomial distribution to detect the methylated cytosine loci. At every detected cytosine loci (${\rm{i}}$), we calculated the probability that methylated reads (${k}_{i}$) occur out of total read number (${n}_{i}$) based on binomial distribution with the success probability (${\rm{p}}$) as bisulfite non-conversion rate. If the p-value was under a certain threshold, we determined the cytosine loci as truly methylated.

2 $$f({k}_{i};{n}_{i},p)=Pr({X}_{i}={k}_{i})=(\begin{array}{c}{n}_{i}\\ {k}_{i}\end{array}){p}^{{k}_{i}}{(1-p)}^{{n}_{i}-{k}_{i}}$$

In addition, we calculated false discovery rate (FDR) to eliminate false positive. We created an artificial methylome for every WGBS sample, in which read depth at each cytosine was equal to real data. Here, methylated read depth was generated following binomial distribution, using the bisulfite non-conversion rate as success probability. We calculated FDR by identifying methylated cytosines in artificial data, and finally set the p-value as 10^−5^, by which the FDR was under 0.01 within all WGBS samples.

### Correlation analysis

We divided whole-genome into 1 k-bp blocks to compare the methylation pattern at CpG and CpH. Then, the correlation between CpG and CpH methylation levels was calculated across blocks that contain more than 10 CpGs and CpHs. In addition, to compare the mCpHs in Cis/Trans-strand to mCpG, we extracted blocks where the difference of mCpG level is over 0.5. Additionally, we measured the correlation between mCpG and mCpH levels in genic regions, using blocks of which more than 500 bp was covered by the genic regions. Genic regions were defined as follows: promoter defined as transcription start site ± 5000 bp, intragenic as all the regions of transcription start site (TSS) to transcription termination site (TTS), and intergenic as complementary of intragenic. The position information of TSS, TTS, and exon regions were obtained from RefSeq annotation. Lastly, to analyse the correlation based on the distance between CpGs and CpHs, we measured average mCpH level of every 10 bp from CpGs, to ±500 bp.

### Gene expression

RNA-seq data were downloaded from NCBI GEO, and pre-processed along the same line with the WGBS data. The pre-processed reads were aligned by Tophat2^48^, and the fragments per kilo-base of transcript per million mapped reads (FPKM) was extracted by Cufflink^49^. In addition, we collected reads per kilobase of transcript per million mapped reads (RPKMs), and FPKMs of DNMT3a and DNMT3b in brains^50^ and ESCs^51–53^, respectively, for statistical analysis (S. Table 2).

### Histone marks

Histone ChIP-seq data were downloaded and mapped into the reference genome by Bowtie^54^. Then, MACS2^55^ was used for peak calling with default parameters (P < 0.00001, mfold > 10). In addition, H3K27ac and H3K4me1 peaks were combined for defining putative enhancer regions. The rate of gene-body region overlapped by each histone mark was calculated as follow.

3 $$histone\,overlapping\,rate=\frac{length\,of\,genebody\,region\,overlapped\,by\,histone\,peaks}{total\,length\,of\,genebody\,region}$$

### Gene ontology analysis

We analysed enriched gene ontology terms (GO terms) to explore approximate function of genes marked by both H3K36me3 and mCAGs. First, we selected 418 genes whose gene-body regions are highly methylated (mCAG level > 0.05, top <3% out of 25919 unduplicated genes), and are enriched with H3K36me3 marks (${histone\; overlapping\; rate}$ > 0.5). Then, GOrilla^56^ was used for identifying enriched GO terms with P-value <10^−3^. The genes and enriched GO terms in HUES64 are described in S. Tables 4 and 5, respectively. Then, we used REVIGO^57^ for clustering GO terms, with similarity >0.5. The specifics for the clustered GO terms are described in S. Table 6. In all steps, we considered genes that length >1000 bp. In addition, gene symbols that start with “LOC” and “Rik” were not included in analysis since those are not recognized by GOrilla.

### Statistics

The Wilcoxon signed rank test were used for determining significance, with P < 0.05. In addition, the Pearson’s correlation coefficient (PCC) was used for measuring correlation. All the boxes in boxplot boxes include values from 25% to 75% in order. The number of data points for drawing Figures and Extended Data Figures are described in S. Tables 4 and 5, respectively.

### Data availability

The data that support the findings of this study are available in Gene Expression Omnibus (GEO). The identifiers for WGBS data are GSE16256^25^, GSE17312^25^, GSE47966^5^, GSE46710^21^, GSE46644^22^, GSE63278^12^, GSE32268^20^, GSE61457^23^, GSE30206^58^, and GSE57413^24^, and those for RNA-seq data are GSE16256^25^, GSE47966^5^, GSE30280^58^, GSE30567^59^, GSE24399^52^, and GSE75748^53^, and those for ChIP-seq data is GSE16256^25^. Statistics of the data are described in S. Tables 1 and S3. In addition, the genome-wide CpG and CpH methylome processed by three bisulfite-read aligners are browsable at Openlooper (https://openlooper.hgc.jp/), with identifiers described in S. Table 1.

## Electronic supplementary material

Supplementary Information

Supplementary Dataset1-7
