# Supplementary material for: Differential landscape of non-CpG methylation in embryonic stem cells and neurons caused by DNMT3s
Source: Sci Rep. 2017 Sep 12;7:11295. doi: 10.1038/s41598-017-11800-1 (PMC5595995; doi:10.1038/s41598-017-11800-1)
Supplement: Supplementary file 1 — Supplementary Information [file 41598_2017_11800_MOESM1_ESM.pdf]

Supplementary Information

Differential landscape of non-CpG methylation in embryonic stem cells and neurons caused by DNMT3s

Jong-Hun Lee[1], Sung-Joon Park[1], and Kenta Nakai[1,*]

Human Genome Center, the Institute of Medical Science, the University of Tokyo, Tokyo, Japan

* Corresponding author
Phone: +81-3-5449-5131
E-mail: knakai@ims.u-tokyo.ac.jp

Supplementary Figures (S. Fig.) 1-7

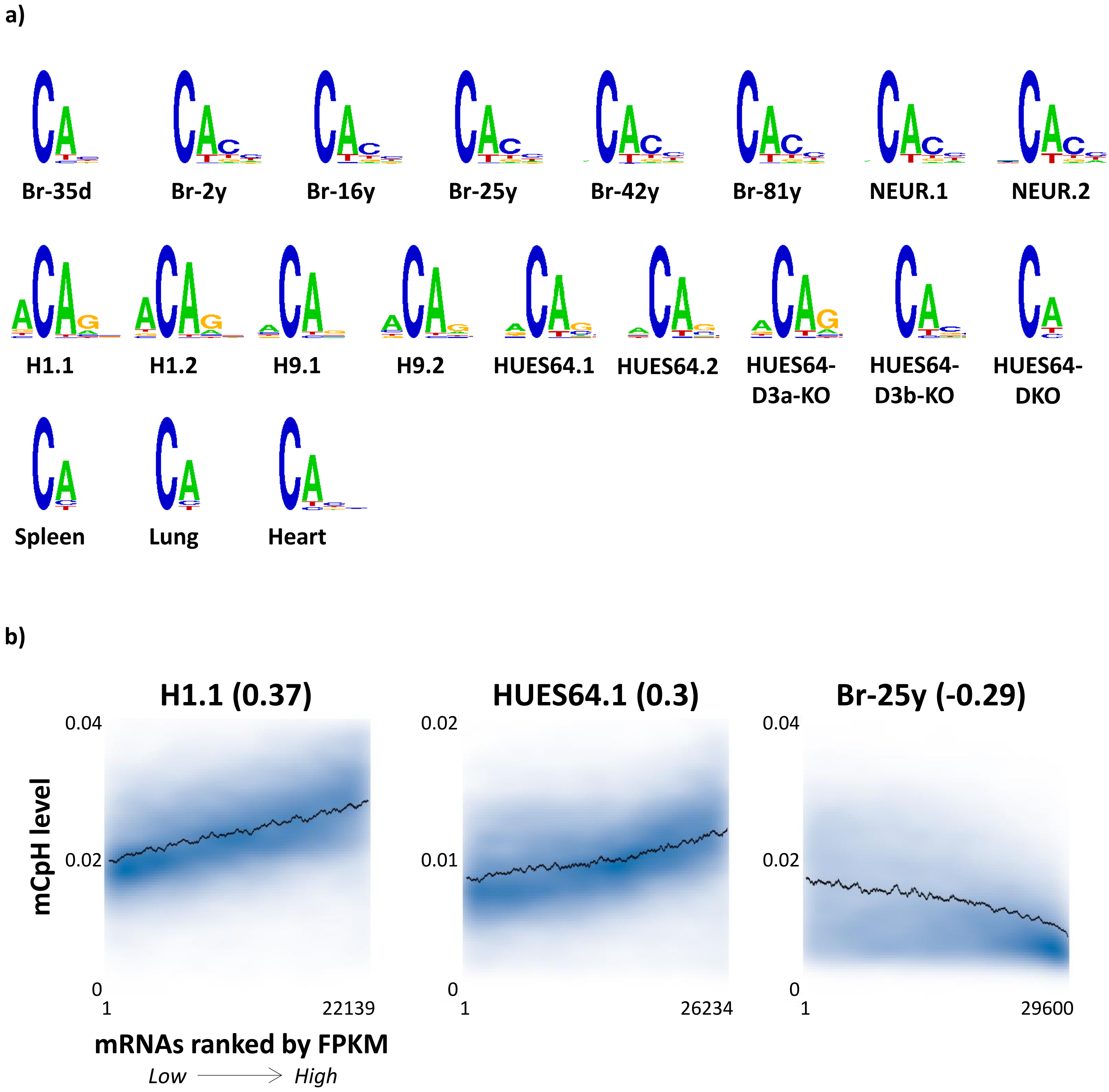

S. Fig. 1| Reproduced differential features of mCpHs between human ESCs and neurons

(a) DNA motifs that abundant near hyper-methylated CpHs (-1bp to +3bp from cytosines at CpH contexts; methylation level (beta value) >0.5), drawn by a software, "weblogo3", in which the height of the characters are defined as the posterior mean relative entropy through Bayesian calculation (default). Br represents brain tissue, and d and y represents days and years, respectively (e.g. Br-2y represents 2-year old brain. NEUR represents neuron. H1, H9, and HUES64 are human ESC cell lines. D3a-KO, D3b-KO, and DKO represents DNMT3a, 3b, and double (3a and 3b) knock out, respectively. (b) Correlation between transcription level of mRNAs (FPKM) and CpH methylation level at their gene-body regions. X-axis is mRNAs ranked by FPKM, with filtering by length>1000, and FPKM>0.1. The parenthesized numbers next to sample names represents Spearman's rank correlation coefficients (ρ) between mCpH level and FPKM. The blue gradation represents density of data points and black line reveals 500-bp window-slided values. The specifics for the WGBS samples and matched RNA-seq samples are described in S. Table 1. This graph is dawn by R package, 'ggplot2'.

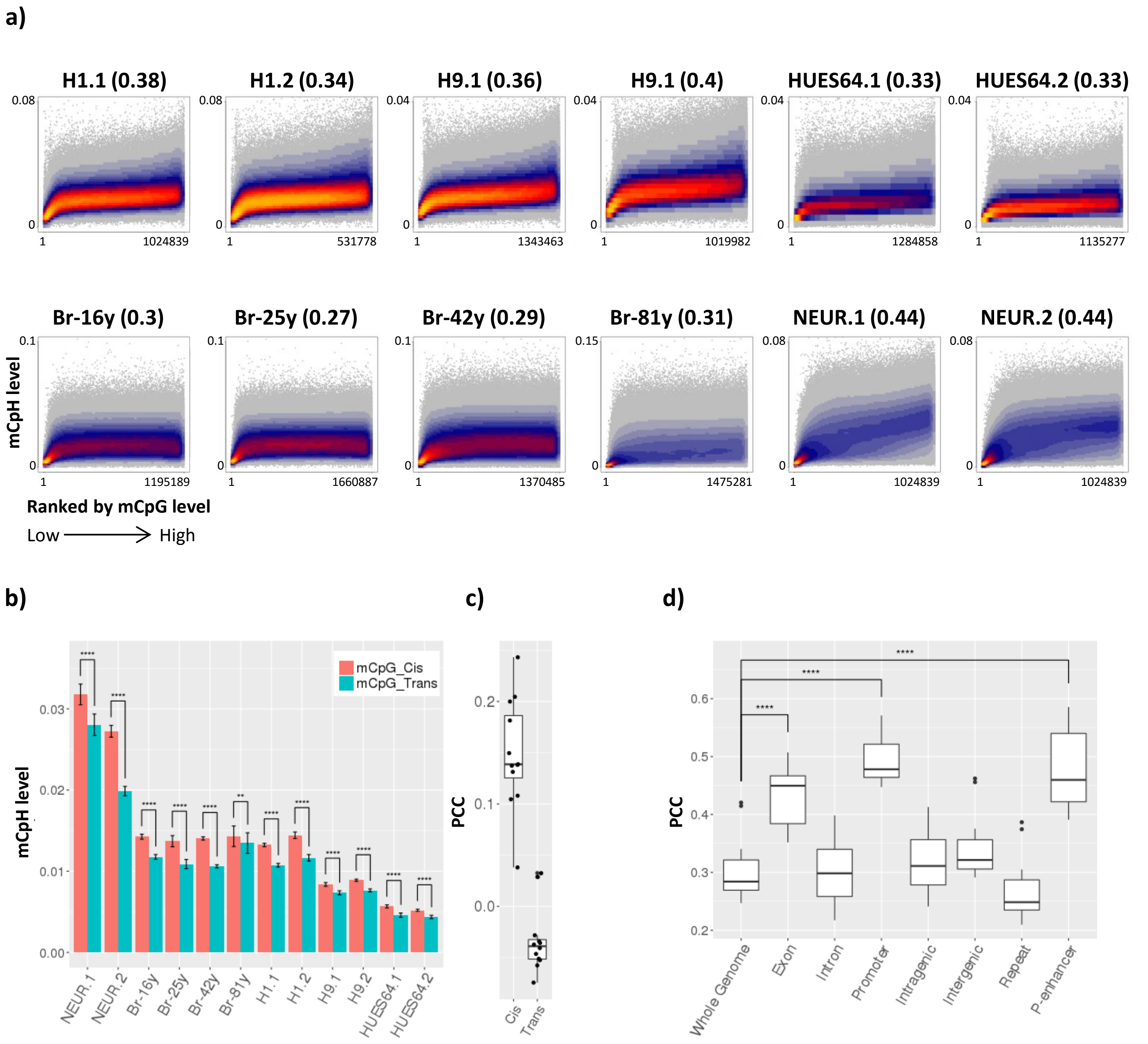

S. Fig. 2| Correlation between CpG and CpH methylation

(a) Scatter plots showing average CpH methylation levels (mCpH level) in 1k-bp blocks that ordered by average CpG methylation level . X-axis shows the number of counted blocks, containing more than 10 CpGs and CpHs. Colors represent density of points; yellow represents the highest density. The parenthesized numbers represent Pearson correlation coefficients between mCpH and mCpG levels across blocks. This graph has been drawn by R package, 'LSD'. (b) Bar graphs showing average mCpH levels in 1k-bp-long blocks where CpGs are hemi-methylated (mCpG level-difference between strands ≧0.5). Error bars represents standard error among the blocks. Stars represent p-values from Wilcoxon Rank Sum test; *,**,***,and **** represent that p-value is under 0.1, 0.01, 0.001, and 0.0001, respectively. (c) Box plot represents Pearson correlation coefficient (PCC) between mCpG and mCpH levels across CpG-hemi-methylated 1k-bp-long blocks in all samples listed in x-axis of graph (b). Cis represents the correlation between mCpG and mCpH in same strand, and Trans represents the correlation between opposite strands. The boxes include values from 25% to 75% in order. (d) The Boxplots consist of the Pearson correlation coefficients (PCC) between mCpG and mCpH levels in each genic region among samples listed in x-axis of graph (b). The correlation coefficients were extracted by comparing blocks that more than 500bp were covered by each genic region. The promoter region is defined as transcription start sites (TSS)±5k-bp, and the Repeat region is extracted from Repeatmasker. In addition, Putative enhancer (P-enhancer) regions are extracted from intersections of two histone mark, H3k27ac, and H3k4me1, detected by MACS2 [62].

a)

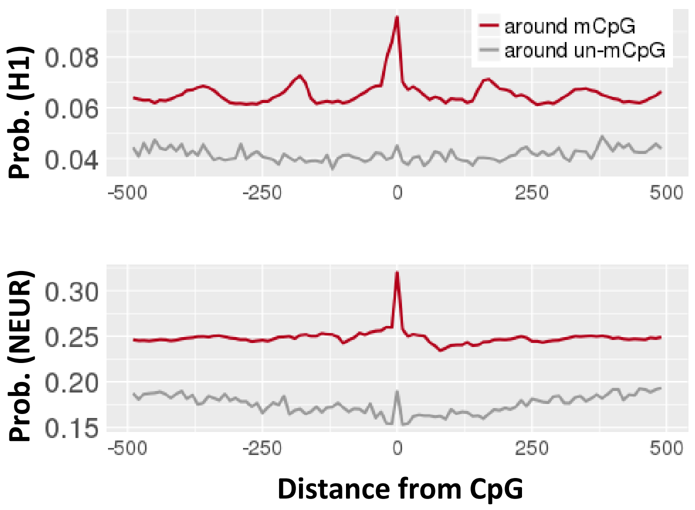

b)

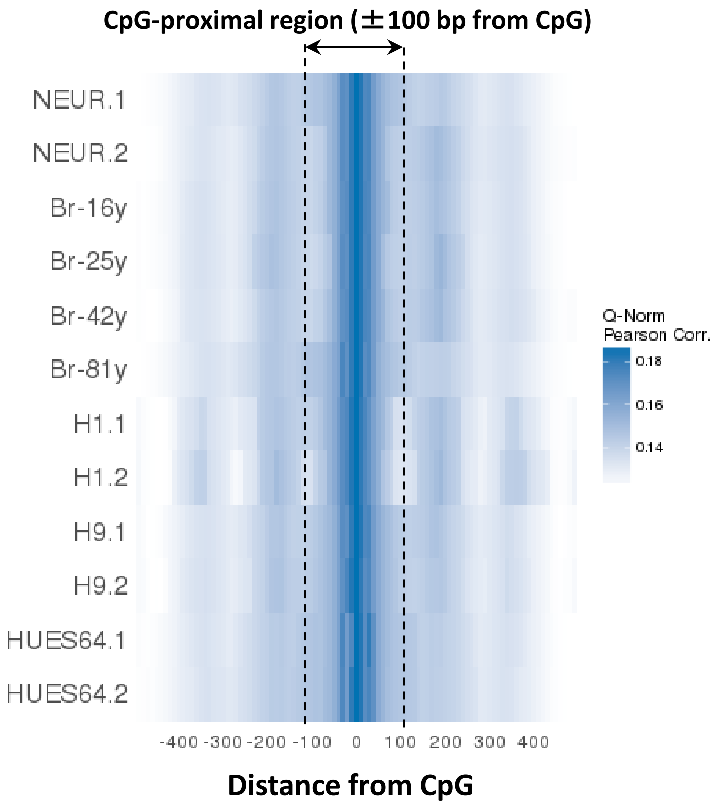

S.Fig. 3| Correlation between mCpGs and flanking mCpHs
(a) Probability that mCpHs exist around (±500 bp) methylated CpGs (red), and un-methylated CpGs (grey), in which no other CpGs exist. To calculate the probabilities, the surrounding (±500 bp) of every CpG divided into 10-bp-long blocks, and blocks containing more than one mCpH were counted, given the condition of CpGs (methylated or un-methylated). (b) Heatmap showing the correlation between mCpG level at every CpGs and surrounding mCpH levels. The surroundings of every CpGs were divided into 50bp-long blocks, then calculated Pearson correlation coefficients (Pearson Corr.) between mCpG levels and mCpH levels of the blocks. To show clear tendency, the correlation coefficients were quantile-normalized (Q-norm). The CpG-proximal region is described with dotted black lines.

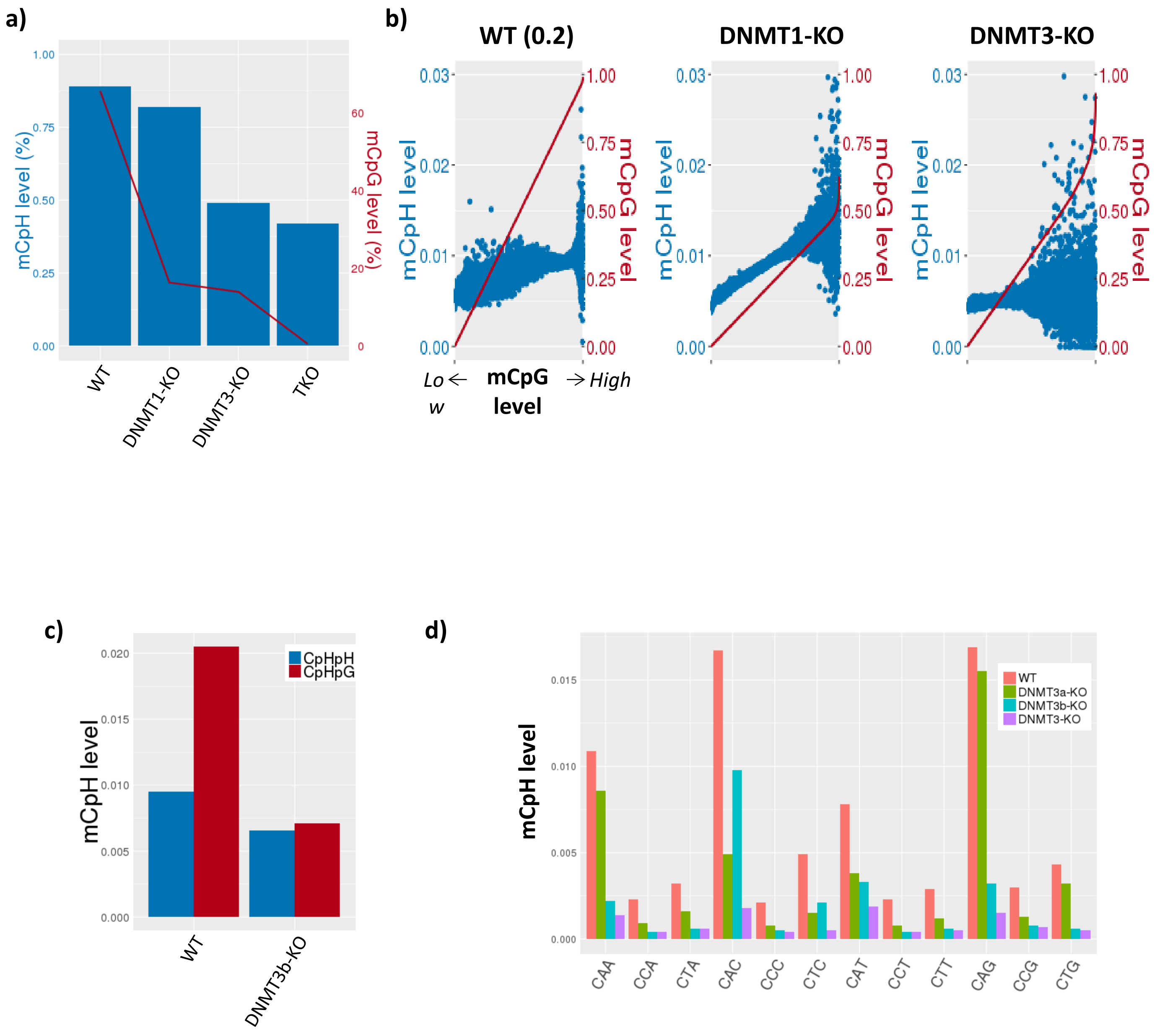

S.Fig. 4| CpH methylation in DNMT knock out human and mouse ESCs

(a) The average mCpG (red line) and mCpH (blue bar) levels in wild type, DNMT1 knock out (DNMT1-KO), DNMT3a/b double knock out (DNMT3-KO), and DNMT1/3a/3b triple knock out (TKO) mouse ESCs. (b) Methylation levels at CpGs (Red lines) and CpHs (Blue dots) in 1k-bp-long blocks that containing more than 10 CpG and CpH points. The blocks revealing same mCpG level were combined and ordered by the mCpG level. The parenthesized numbers beside sample names represents Pearson correlation coefficients between mCpG and mCpH levels. (c) Average CpH methylation levels at CpHpH (blue) and CpHpG (red) contexts in wild type and DNMT3b knock out human ESCs (H9.1 sample). (d) Average methylation level at CpHpNs (N means all nucleotides) in HUES64.

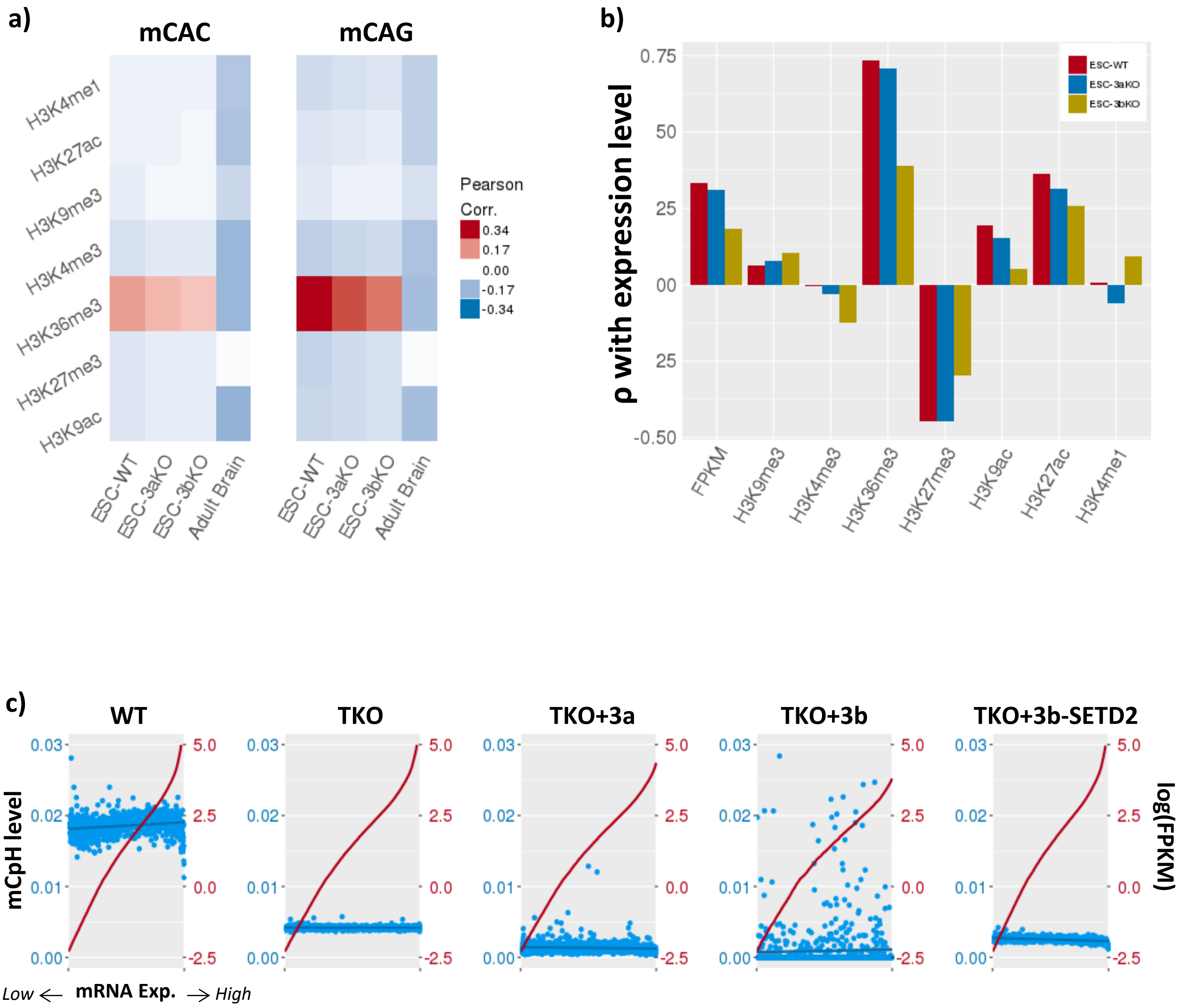

S.Fig. 5| CpH methylation in gene-body regions and H3K36me3 marks

(a) Correlation between histone marks in wild type HUES64 (WT) with methylation levels at CACs (left) and CAGs (right) in wild type, DNMT3a-knock out, and DNMT3b-knock out HUES64s (ESC-WT, -3aKO, and -3bKO, respectively), and adult brain (25-year old). (b) Spearman's rank correlation coefficient (ρ) between mCpH level with expression level (FPKM) and Histone marks (x-axis) in gene body regions. (c) Gene expression levels (FPKM) of wild-type mouse ESC, and CpH methylation levels in gene-body regions of wild-type (WT), DNMT1/3a/3b knockout (TKO), DNMT3a re-induced TKO (TKO+3a), DNMT3b re-induced TKO (TKO+3b), and SETD2-knocked out from TKO+3b sample (TKO+3b-SETD2) mouse ESCs. Genes are selected by length > 1k-bp, and FPKM>0.1. For visualisation, we ordered the mRNAs by FPKM value and represented 1000 data points, averaged mCpH levels of genes grouped by expression levels. Linearly smoothed line (blue line) and 95% confidence interval (grey zone) of mCpH levels are represented.

a)

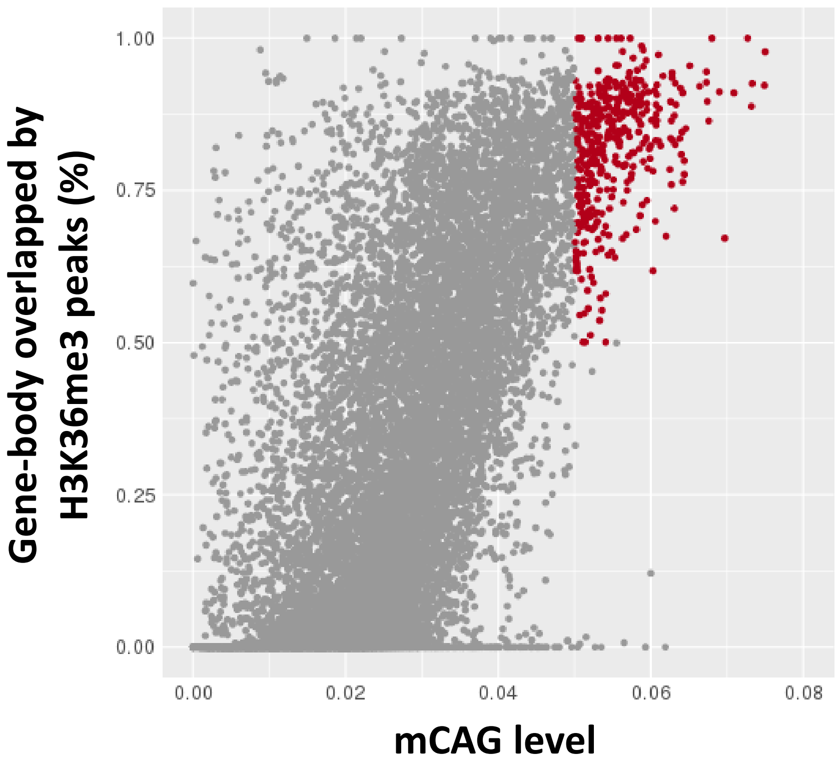

b)

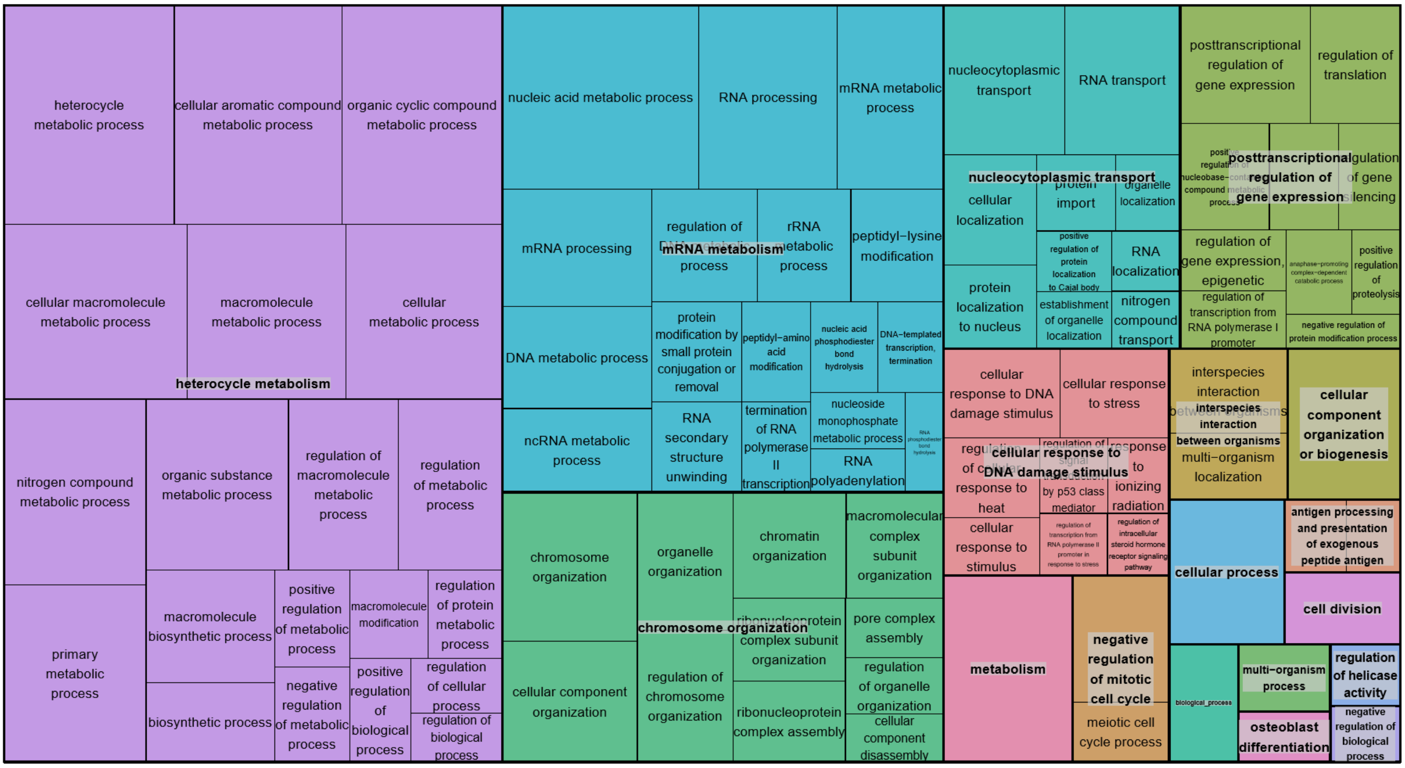

S.Fig. 6| Screening genes marked by both hyper-mCAG and H3K36me3

(a) Average methylation level at CAGs (x-axis) and H3K36me3-overlapping rate (y-axis) in gene-body regions of 25919 genes (length>1000bp) in wild type HUES64. Red dots represents the selected geneset filtered by mCAG level > 0.05, and H3K36me3-overlapping rate > 0.5. Values are described in S. Table 7 (b) Clustered GO terms enriched with the geneset. The enriched GO terms from biological processes (BP) (P-value<0.001) were clustered by similarity>0.5. The GO terms are clustered and drawn as treemap by REVIGO [57].

ADAR

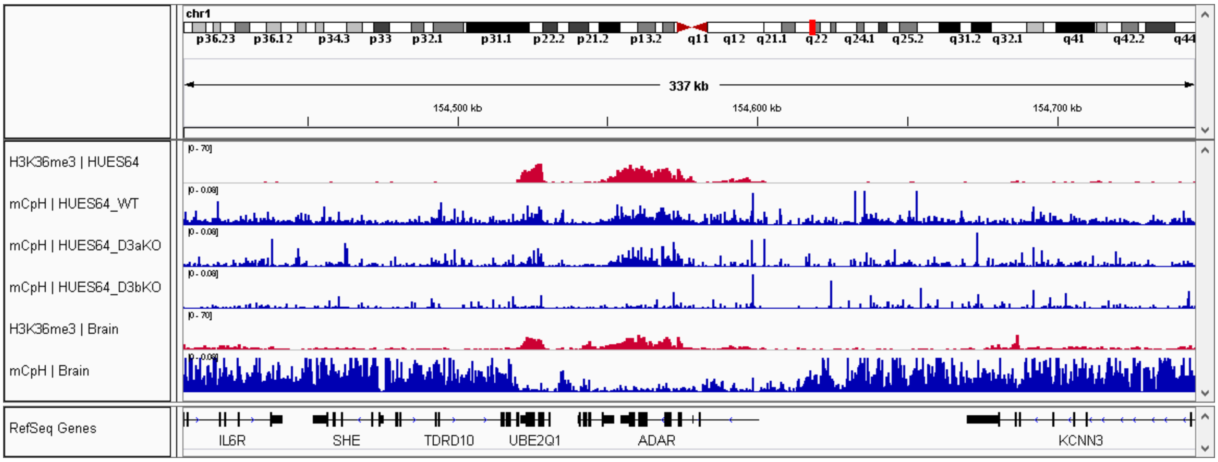

CCNB1

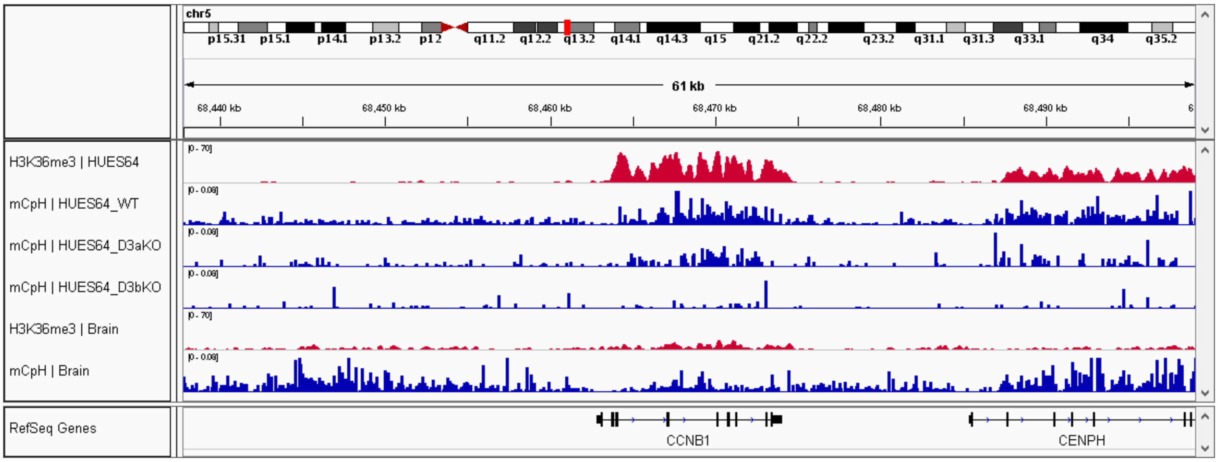

MYH10

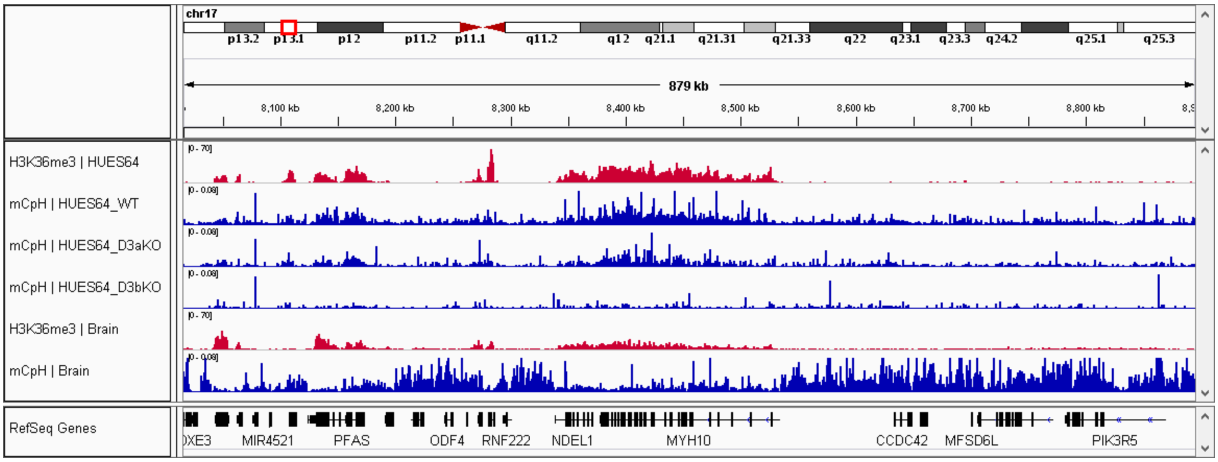

S.Fig. 7| Examples of genes related to embryo development

The screenshots represent H3K36me3 peaks (red bars) and mCpH level (blue bars) upon embryo development-related genes, ADAR, CCNB1, and MYH10. These are drawn by IGV [61].
